# Data-driven analysis and prediction of dynamic postprandial metabolic response to multiple dietary challenges using dynamic mode decomposition

**DOI:** 10.3389/fnut.2023.1304540

**Published:** 2024-01-12

**Authors:** Viktor Skantze, Mats Jirstrand, Carl Brunius, Ann-Sofie Sandberg, Rikard Landberg, Mikael Wallman

**Affiliations:** ^1^Fraunhofer-Chalmers Research Centre for Industrial Mathematics, Gothenburg, Sweden; ^2^Department of Life Sciences, Division of Food and Nutrition Science, Chalmers University of Technology, Gothenburg, Sweden

**Keywords:** personalized nutrition, differential responders, metabotypes, dynamic mode decomposition, precision nutrition

## Abstract

**Motivation:**

In the field of precision nutrition, predicting metabolic response to diet and identifying groups of differential responders are two highly desirable steps toward developing tailored dietary strategies. However, data analysis tools are currently lacking, especially for complex settings such as crossover studies with repeated measures.

Current methods of analysis often rely on matrix or tensor decompositions, which are well suited for identifying differential responders but lacking in predictive power, or on dynamical systems modeling, which may be used for prediction but typically requires detailed mechanistic knowledge of the system under study. To remedy these shortcomings, we explored dynamic mode decomposition (DMD), which is a recent, data-driven method for deriving low-rank linear dynamical systems from high dimensional data.

Combining the two recent developments “parametric DMD” (pDMD) and “DMD with control” (DMDc) enabled us to (i) integrate multiple dietary challenges, (ii) predict the dynamic response in all measured metabolites to new diets from only the metabolite baseline and dietary input, and (iii) identify inter-individual metabolic differences, i.e., metabotypes. To our knowledge, this is the first time DMD has been applied to analyze time-resolved metabolomics data.

**Results:**

We demonstrate the potential of pDMDc in a crossover study setting. We could predict the metabolite response to unseen dietary exposures on both measured (*R*^2^ = 0.40) and simulated data of increasing size ($R_{\text{max}}^{2}$= 0.65), as well as recover clusters of dynamic metabolite responses. We conclude that this method has potential for applications in personalized nutrition and could be useful in guiding metabolite response to target levels.

**Availability and implementation:**

The measured data analyzed in this study can be provided upon reasonable request. The simulated data along with a MATLAB implementation of pDMDc is available at https://github.com/FraunhoferChalmersCentre/pDMDc.

## 1 Introduction

Diet is one of the main modifiable lifestyle factors that contribute to health (1). It is known that individuals exhibit large differences in response to diet and nutritional requirements, which motivates the research in precision nutrition (PN) for improved population health (2–5). PN can be defined as providing the right diet to the right person at the right time, i.e., optimizing the dietary intake for individual needs, which requires prediction of individual responses to diet.

Prediction of metabolite response is still considered a difficult problem, since no mechanistic model as of yet covers the entire human metabolome, although important progress has been made in recent years. In a landmark study by Zeevi et al. (6), individual postprandial glycemic responses to various foods were successfully predicted from blood parameters, dietary habits, anthropometrics, physical activity, and gut microbiota highlighting the potential for personalized dietary recommendations to lower glycemic response. Later, in the PREDICT 1 study, meal composition, habitual diet, meal context, anthropometry, genetics, microbiome, clinical and biochemical parameters were used to predict individual postprandial responses in triglycerides, glucose, and insulin successfully to responses of food intake (7). Notably, both of these studies targeted only a small number of metabolites (one and three respectively) which are determinants for cardiometabolic diseases. However, many more metabolites have been associated with health and disease outcomes, and it will become a desirable trait in PN to guide a larger part of the metabolite levels to optimize response to, e.g., dietary exposures (8). Wang et al. recently used dietary data and microbiome to predict metabolome profiles at a static point in time using deep learning (9, 10). However, deep neural network architectures are often challenging to interpret, which is desirable when connecting dietary contents and metabolite response. Furthermore, prediction of metabolite response over time will provide more information than single time points. However, such high-dimensional multivariate time series forecasting has not yet been explored in PN (11).

Rather than focusing on individual metabolite responses, an alternative approach within PN is to identify groups of individuals with similar latent metabolic phenotypes (i.e., metabotypes) and tailor dietary advice for the group (1, 12, 13). Identifying metabotypes relies heavily on dimensionality reduction to capture essential trends in the data that can subsequently be clustered. This has previously been done in matrix (single point in time) and multiway/tensor metabolomics data (time series) using matrix and tensor decomposition methods respectively, such as principal component analysis (PCA) and CANDECOMP/PARAFAC (CP) (14, 15). Tensor decomposition methods like CP are useful for interpreting time series data in, e.g., metabolomics but are descriptive methods and not inherently predictive. Furthermore, CP which arguably is the more interpretable tensor decomposition (compared to, e.g., Tucker decomposition), has a multi-linear structure that does not fit all tensor data, leaving unexplained variance in the data.

Addressing both the problem of prediction and dimensionality reduction, dynamical mode decomposition (DMD) (16) has recently emerged as a promising tool for analysis of large-scale dynamical data. However, to the best of our knowledge, the usefulness of DMD has not yet been investigated in the analysis of time-resolved metabolite data. DMD works by identifying a low-rank linear dynamical system (LDS, a linear state space model) directly from data. The low-rank LDS captures the “essential” dynamics underlying the full system under study, and mapping between the low-rank system and the full system can be performed using a linear operator. Thus, DMD naturally incorporates both prediction (in the form of dynamical modeling) and dimensionality reduction (in the reduced rank of the model), and thus holds the potential to become a valuable tool within PN. Further, LDSs are well-studied, interpretable, and have been used extensively to model and forecast phenomena in a wide variety of research fields (17). Moreover, these systems come with a range of analytical perks such as stability and identifiability analysis, automatic control, and analytical state solutions (18).

Since its introduction, several variants of DMD have been proposed (19–23). Notably, parametric DMD (pDMD) allows several perturbations of the same system to be used to infer the parameters of a single DMD model (24). This makes it well suited to infer the metabolic system by integrating data from, e.g., different intervention diets. In addition, another development denoted DMD with control (DMDc) (22) allows incorporation of system input to the identified LDS, which is well aligned with, e.g., dietary interventions to separate the metabolic regulation from the dietary exposures.

Our aim was therefore to explore the use of DMD for the analysis of time-resolved metabolomics tensor data. Specifically, we employ a combination of pDMD with DMDc (denoted pDMDc) to predict the metabolite response of a given individual to new diets after having trained on several others, and to identify potential metabotypes from LDS trajectories. As a realistic setting for this method development, we used measured metabolomic data from a dietary intervention crossover study, with variability in four dimensions: metabolite, time, individual, and diet. The methodology was further evaluated using identically structured synthetic data from a large human metabolome model (25), containing two clusters of individuals (healthy and diabetic) used as ground truth for metabotyping, and responses from simulated dietary interventions as ground truth for prediction. The identification of potential metabotypes was performed by clustering latent dynamic states derived from pDMDc using measured and simulated data, and compared to clustering scores derived using CP.

## 2 Materials and methods

### 2.1 Data and workflow overview

#### 2.1.1 Measured data from a dietary intervention study

Time-resolved metabolite data were obtained from a dietary crossover intervention trial (26). The trial comprised 17 middle-aged overweight men (BMI 25–30 𝑘𝑔/𝑚^2^, 41–67 years of age), all consuming three different diets (pickled herring, baked beef, and baked herring) on separate test occasions, as detailed in Table 1.

**Table 1:** Macronutrients of the intervention diets.

Meal	Fat, *g*	Protein, *g*	Carbohydrate, *g*
Baked herring	29	33	47
Pickled herring	29	29	81
Baked beef	35	43	47

**Figure 1:**
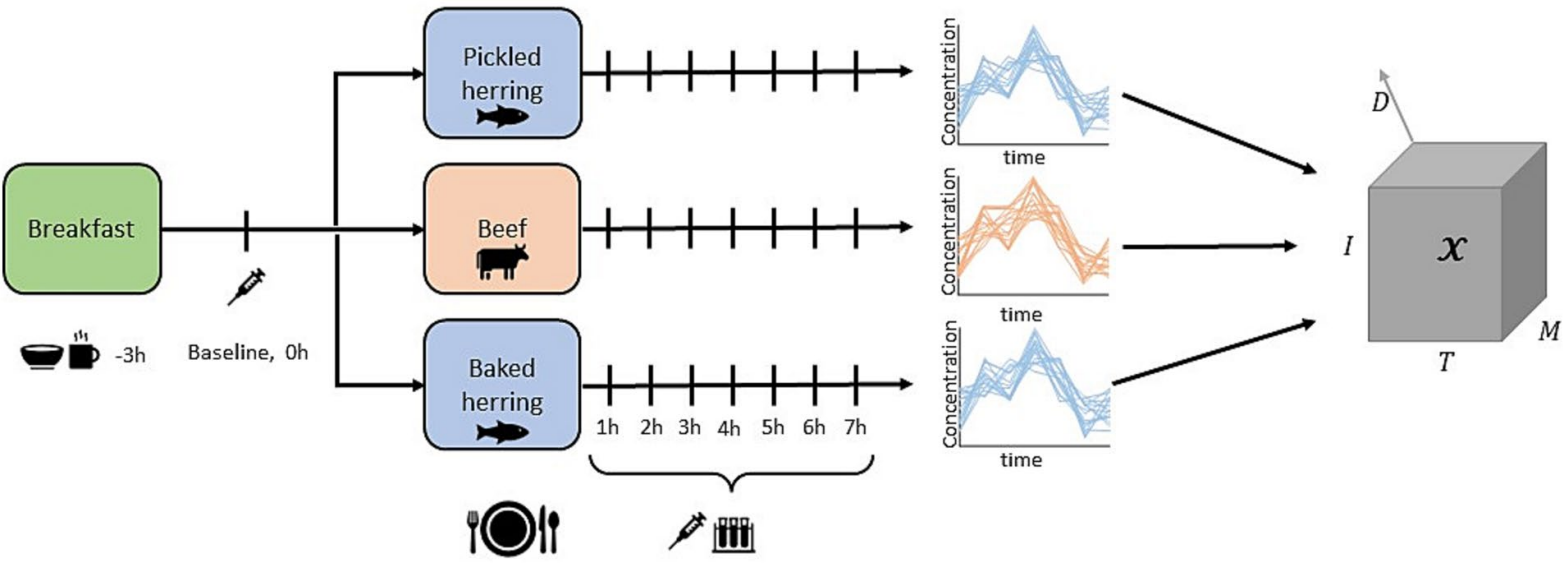
Study design graphic of the three-armed crossover dietary interventional study.

Baseline clinical measures along with anthropometric measures were recorded, including alanine aminotransferase (ALAT), aspartate transaminase (AST), gamma-glutamyl transferase (GGT), cholesterol, low-density lipoprotein (LDL), creatinine, thyroid stimulating hormone (TSH), and body mass index (BMI). On each test occasion, blood samples for metabolomics analysis were taken 8 times at one-hour intervals, including a baseline sample just before the meal was consumed. Metabolomics analysis was performed using GC-MS and 79 targeted metabolites were measured, resulting in 79 metabolite trajectories with 8 time points for three different diets for each individual. The main categories of metabolites contained in the data were: amino acids (*n* = 35), carboxyl acids (*n* = 6), lipids (*n* = 8), and carbohydrates (*n* = 16). The data can be viewed as a fourth-order tensor (four-way array) $X\in R^{M\times T\times I\times D}$ with M=79 metabolites, T=8 time points, I=17 individuals and D=3 diets. A graphical overview of the study design and experimental data is presented in Figure 1. The baseline measurement, $x_{m,t=0,i,d}$, was subtracted from each time series to focus the analysis on the postprandial dynamics in the data from the study intervention. For proper values on evaluation metric (*R*^2^) we standardized the response per diet to unit variance.

#### 2.1.2 Simulated data from a virtual metabolic human dynamic model

Data was simulated for two purposes; (i) to evaluate prediction performance of response to unseen dietary exposures using pDMDc, and (ii) to evaluate metabotyping with ground truth clusters using pDMDc and CP. We used a virtual metabolic human dynamic model for pathological analysis (25) to generate simulated data. The model consists of 202 ordinary differential equations modelling the human metabolome using 1,140 kinetic parameters. Inputs to the model are amounts and uptake rates of glucose and triglycerides, and the outputs are dynamic postprandial responses of 202 metabolites.

For the dietary response prediction study, we generated a dataset consisting of 17 healthy simulated individuals (same number as the measured data in Section 2.1.1) and 90 postprandial responses to different dietary exposures. For the metabotyping study, data from 50 healthy and 50 diabetic individuals were generated as postprandial responses to 3 meal interventions. The meals were generated by sampling the glucose and triglyceride content and their uptake rates from a normal distribution, as detailed in Table 2, “dietary parameters.” Differences in individual metabolism were modelled using a selection of kinetic parameters impacting the diabetic characteristic, as detailed in Kurata (25), with mean and standard deviations set to produce typical responses from healthy and diabetic individuals (Table 2, “individual parameters”). Initial values for the simulations were produced by running the model with the default food input (mean values of the dietary parameters in Table 2) for 10 h.

**Table 2:** Individual parameters: description of parameter distributions for simulated postprandial responses of diabetic and healthy individuals, drawn from normal distributions with the indicated mean and standard deviation.

Type	Variable	Description	Value health (mean ± std)	Value diabetic (mean ± std)	Unit
Individual parameters	$Vmax_{\mathrm{lipog}\;l}^{\mathrm{Acco}a_{LC}}$	Lipogenesis rate for AcCoa in liver	0.12 ± 0.0192	0.24 ± 0.0384	mmol/min
	$Vmax_{\mathrm{lipog}\;2}^{\mathrm{Malco}a_{LC}}$	Lipogenesis rate for Malcoa in liver	1 ± 0.16	2 ± 0.32	mmol/min
	$Vmax_{\mathrm{tgsyn}}^{FFA_{L}}$	TG synthesis rate for FFA in liver	0.4 ± 0.0640	1.6 ± 0.256	mmol/min
	$Vmax_{\mathrm{cholsynl}}^{\mathrm{Acco}a_{LC}}$	cholesterol synthesis rate for AcCoa in liver	0.02 ± 0.0032	0.04 ± 0.0064	mmol/min
	$Km_{\mathrm{inssyn}}^{\mathrm{Gl}c_{B}}$	MMc for insulin synthesis	7.5 ± 1.2	11.25 ± 1.8	mmol
	$Km^{\mathrm{In}s_{BL}}$	MMc for insulin in liver	(1 ± 0.16)·10^−8^	(1.5 ± 0.24)·10^−8^	mM
	$Km^{\mathrm{In}s_{BM}}$	MMc for insulin in skeletal muscle	(1 ± 0.16)·10^−8^	(1.5 ± 0.24)·10^−8^	mM
	$Km^{\mathrm{In}s_{\mathrm{BA}}}$	MMc for insulin in adipose tissue	(1 ± 0.16)·10^−8^	(1.5 ± 0.24)·10^−8^	mM
Meal parameters	$\mathrm{Gl}c_{B}^{\mathrm{meal}}$	total input glucose	220 ± 88.9	220 ± 88.9	mmol
	$T_{\mathrm{delay}}^{Glc}$	time delay of glucose release	50 ± 20.2	50 ± 20.2	min
	$TG_{B}^{\mathrm{meal}}$	total input TG	25 ± 10.1	25 ± 10.1	mmol
	$T_{\mathrm{delay}}^{TG}$	time delay of TG release	240 ± 96.9	240 ± 96.9	min

Meal parameters: the four parameters governing the input to the simulated metabolic system, generating different diets from the normal distribution. The values are indicated in mean and standard deviation. MMc stands for Michaelis–Menten constant.

For each simulated dataset, the model simulated metabolite dynamics for 10 h, producing 60 time points sampled at 10-min intervals. Logarithmic down-sampling resulting in eight time points was done to make the simulated data more similar to the measured data and to save computing time when performing DMD. Metabolite concentrations with a standard deviation less than 10^−3^ mM during the course of the simulation were excluded, leaving 130 dynamic metabolites for further analysis. When computing the *R*^2^ values, we standardized the response per diet to unit variance.

### 2.2 Linear dynamical systems

#### 2.2.1 Discrete linear dynamical systems

In this work, we describe the dynamics of metabolite responses using discrete time LDSs, defined by a coupled system of linear difference equations:


(1) $$x_{t+1}=A\;x_{t}+B\;z_{t}$$

Here, *t* is the time index, $x_{t}\in R^{M}$ is a vector of M metabolite concentrations at time *t,* $A\in R^{M\times M}$ is the system matrix representing the best-fit (smallest Frobenius norm) mapping between metabolite time points, $z_{t}\in R^{l}$ is a vector of l system inputs, $B\in R^{M\times l}$ is the best-fit mapping from the input to the metabolite dynamics. We also assume that the measured metabolites can be projected linearly (via the mapping $\tilde{U}\in R^{M\times S}$) to a smaller set of latent dynamical state variables ${\tilde{x}}_{t}\in R^{S}$ of size S (potentially simplifying system analysis), representing the “essential” dynamics of the system, as formalized in Equations (2a, 2b)


(2a) $${\tilde{x}}_{t+1}=\tilde{A}\;{\tilde{x}}_{t}+\tilde{B}\;z_{t}$$

(2b) $${\hat{x}}_{t}=\tilde{U}\;{\tilde{x}}_{t}$$

Here, $\tilde{A}\in R^{S\times S}$ is the reduced system matrix, $\tilde{B}\in R^{S\times l}$ is the mapping from the input to the metabolite dynamics, and ${\hat{x}}_{t}\in {\mathbb{R}}^{M}$ is the approximation of the data consisting of M metabolites. Given these model structures, it remains to infer the dimensionality and parameters of Equations (1, 2a, 2b) from the available data, which we do using DMD.

#### 2.2.2 Conceptual description of the dynamic model

DMD is a method that learns a linear kinetic model structure, along with its parameters, from dynamical data, without any prior knowledge of the underlying system. As integral part, DMD also provides dimension reduction of the data, which in turn reduces the complexity of the learned model. In practice, this means that many correlated measured signals can be represented by a single model state, similar to PCA.

Data that measure the response of a system to some perturbation are suitable for the application of DMD. This fits well with the setting of this study, where the data consist of postprandial responses, measured as time-resolved omics features. The DMD model, representing the metabolic system, is learned using least squares regression, which also has the advantage that optimization of model parameters can be done analytically. This avoids the risk of finding local optima, which is a problem for many machine learning methods (27). Furthermore, the least squares solution can be calculated using the singular value decomposition (SVD), which also allows for dimension reduction and therefore reduction in model complexity.

Additionally, DMD can be extended to include a known input to the model (dynamic mode decomposition with control, DMDc). In this work, the model structure expressed in Equations (2a, 2b) is learned using DMDc, with dietary intake as input and measured postprandial response, in the form of high dimensional metabolomics time trajectories, as output. Furthermore, to include information about responses to different meals, an additional extension called parametric DMD (pDMD) can be combined with DMDc, resulting in the proposed pDMDc method. This development enables prediction of postprandial response to new diets. Since the dimensionality reduction property of DMD still holds for pDMDc, it also allows for identifying the differences in response between individuals in large groups of omics features.

**Figure 2:**
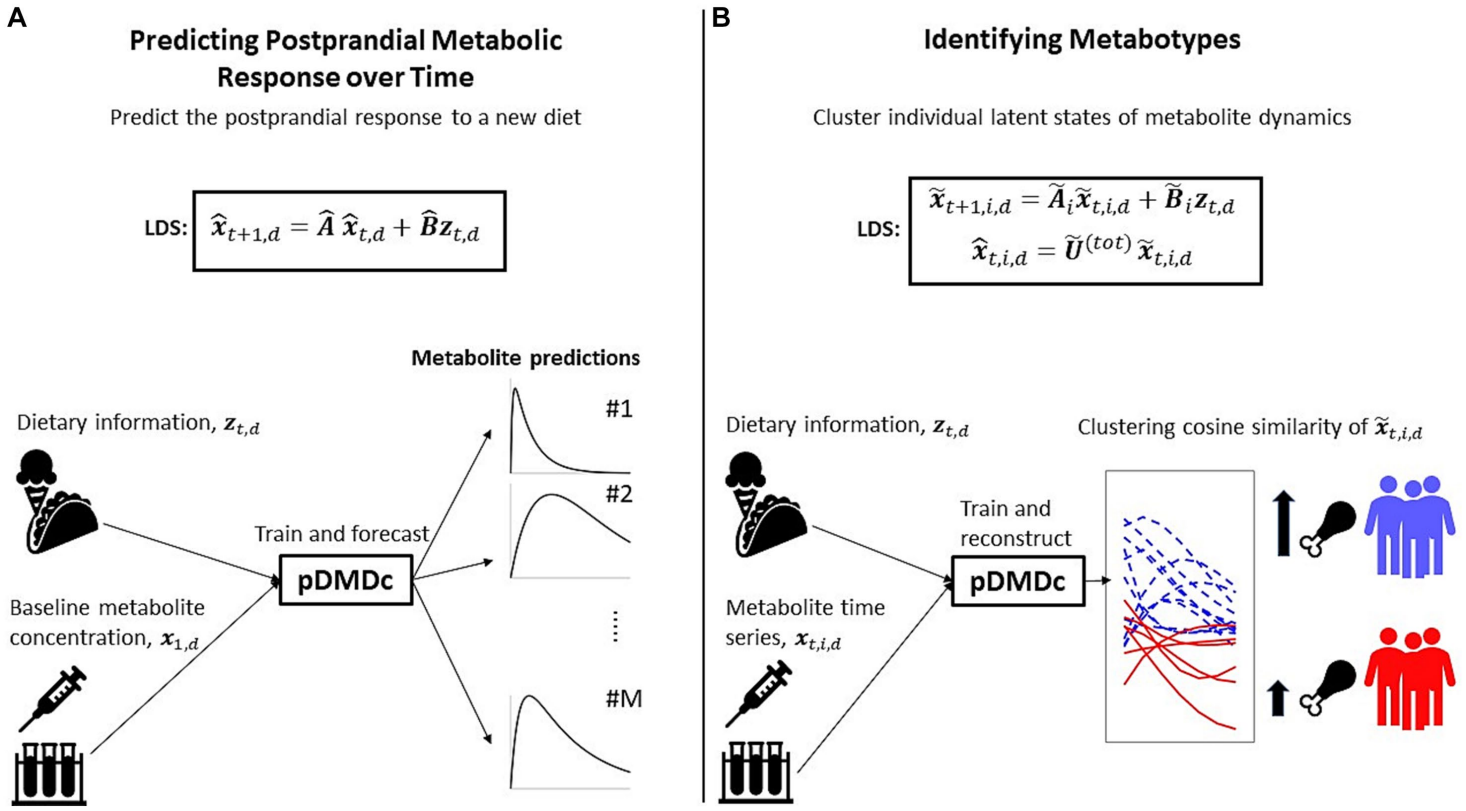
Overview of the two applications for which we use our method. **(A)** Identification of a discrete linear dynamical system (LDS) using parametric DMD with control (pDMDc) allows for prediction of postprandial response of a new diet having trained on several others (Section 2.5). **(B)** Using pDMDc with a shared output mapping ${\tilde{U}}^{\left(\mathrm{tot}\right)}$ that links individuals in the population to the same LDS framework, we cluster latent state trajectories ${\tilde{x}}_{t,i,d}$ to identify metabotypes (Section 2.6).

A mathematical description of DMDc and pDMDc can be found in Sections 2.3 and 2.4, respectively. To give an overview of our proposed data analysis workflow, we summarize the two different applications, i.e., predicting the postprandial metabolite response and the identification of metabotypes, in Figure 2.

### 2.3 Dynamic mode decomposition with control

In the context of the dynamic metabolite measurements described in Section 2.1, vectors of metabolite samples $x_{t}\in R^{M}$ per time point t=1,2,…,T are stored column-wise in the matrices (commonly called snapshot matrices in the DMD literature),


(3) $$X:=\left[\begin{array}{cccc} | & | & & |\\ x_{1} & x_{2} & \ldots & x_{T-1}\\ | & | & & | \end{array}\right]\in {\mathbb{R}}^{M\times \left(T-1\right)}$$

(4) $$X^{\prime }:=\left[\begin{array}{cccc} | & | & & |\\ x_{2} & x_{3} & \ldots & x_{T}\\ | & | & & | \end{array}\right]\in {\mathbb{R}}^{M\times \left(T-1\right)}$$

Thus, one snapshot matrix contains the first T−1 measurements and the other one contains the last T−1 measurements. Note that here we assume that the metabolite measurements stem from a single dietary provocation to the metabolic system.

Further, we assume that the measurements in each time point have been generated from a linear time-invariant system (Equation 1), where each time point is a linear function of the previous measurement and a previous control/input signal. Using the example data, the input $z_{t}$ could represent, e.g., nutrient composition in a dietary intervention, and the data $x_{t}$ represent the postprandial metabolite responses to the intervention. To estimate the matrices A and B, we first construct the control input snapshot matrix, in Equation 5.


(5) $$Z:=\left[\begin{array}{cccc} | & | & & |\\ z_{1} & z_{2} & \ldots & z_{T-1}\\ | & | & & | \end{array}\right]\in {\mathbb{R}}^{l\times \left(T-1\right)}$$

These definitions allow us to formulate an equation analogous to Equation (1) but in full matrix form:


(6) $$X^{\prime }=\mathrm{AX}+BZ$$

Here, $X^{\prime }$, X and Z are already known, so in order to solve for A and B, we restate Equation 6 as


(7) $$X^{\prime }=\left[A\;B\right]\left[\begin{array}{c} X\\ Z \end{array}\right]=G\;\Omega$$

We model the dietary input (represented by the nutritional composition of the intervention diet) as an impulse of size l to the metabolic system at the first time point, which implies that Z is set to zero everywhere apart from the first time point ($u_{1}$). Accordingly, the matrix Σ can be written as


(8) $$\Omega =\left[\begin{array}{ccccc} | & | & | & & |\\ x_{1} & x_{2} & x_{3} & \ldots & x_{T-1}\\ | & | & | & & |\\ z_{1} & 0 & 0 & \ldots & 0 \end{array}\right]$$

We now seek the best-fit solution, in the Frobenius sense, of the operator G, representing a column-wise concatenation of A and B. This is achieved using the Moore-Penrose inverse (denoted †) via the SVD of $\Omega =U\;\Sigma \;W^{⊺}\text{.}$ As commonly done in DMD, we can also choose to truncate the SVD to V ≤ $\mathrm{rank}\left(\Omega \right)$≤ $\left(M+l\right)$ components (e.g., to reduce overfitting and sensitivity to noise), resulting in the reduced matrices $\hat{U}\in {\mathbb{R}}^{\left(M+l\right)\times V}$, $\hat{W}\in {\mathbb{R}}^{\left(T-1\right)\times V}$ and $\hat{\Sigma }\in {\mathbb{R}}^{V\times V}$. Applying the pseudo-inverse and truncated SVD to solve Equation 7 gives us the matrix G as


(9) $$G=X^{\prime }\;\Omega^{†}=X^{\prime }{\left(U\;\Sigma \;W^{⊺}\right)}^{†}\approx X^{\prime }{\left(\hat{U}\;\hat{\sum }\;{\hat{W}}^{⊺}\right)}^{†}=X^{\prime }\;\hat{W}\;{\hat{\sum }}^{-1}\;{\hat{U}}^{⊺}$$

In the text below, $\hat{A}\in {\mathbb{R}}^{M\times M}$and $\hat{B}\in {\mathbb{R}}^{M\times l}$ will be used to denote rank-reduced versions of A and B. The resulting rank-reduced system retains the same size as the original system (Equation 1) but is less prone to overfitting to the given data. We now retrieve the approximations $\hat{A}$ and $\hat{B}$of the two linear operators A and B by dividing G into two parts:


(10) $$\begin{array}{l} \left[\begin{array}{cc} A & B \end{array}\right]\approx \left[\begin{array}{cc} \hat{A} & \hat{B} \end{array}\right]\\ \;=\left[X^{\prime }\;\hat{W}\;{\hat{\sum }}^{-1}\;{\hat{U}}^{\left(X\right)⊺}\;X^{\prime }\;\hat{W}\;{\hat{\sum }}^{-1}\;{\hat{U}}^{\left(z\right)⊺}\right] \end{array}$$

Here ${\hat{U}}^{\left(X\right)⊺}\in {\mathbb{R}}^{M\times V}$ and ${\hat{U}}^{\left(z\right)⊺}\in {\mathbb{R}}^{l\times V}$ represent the bases for the metabolomics data and the dietary input, respectively, as ${\hat{U}}^{⊺}={\left[\;{\hat{U}}^{\left(X\right)⊺}\;{\hat{U}}^{\left(z\right)⊺}\right]}^{⊺}\text{.}$ This yields the rank-reduced system


(11) $${\hat{x}}_{t+1}=\hat{A}\;{\hat{x}}_{t}+\hat{B}\;z_{t}$$

where ${\hat{x}}_{t}$ denotes the approximation of the metabolite concentration $x_{t}\text{.}$

At this stage, the system still represents the dynamics of each metabolite with a separate state. However, for simplified analysis we also aim to reduce the number of states of our LDS to a latent space representation with *S* ≤ *M* states (*cf.* Equations (2a, 2b)). The choice of *S* and *V* is dependent on the data and can be set equal to each other. To do this, we start with the SVD $X^{\prime }=U^{\prime }\Sigma^{\prime }W^{\prime ⊺}$. Next, we truncate the matrix $U^{\prime }\in R^{M\times M}$ to form $\tilde{U}\in R^{M\times S}$, which we use to map the system onto the latent state space. The resulting system matrix, input matrix, and state vector for the reduced order LDS become


(12a) $$\tilde{A}={\tilde{U}}^{⊺}\;\hat{A}\tilde{U}$$

(12b) $$\tilde{B}={\tilde{U}}^{⊺}\hat{B}$$

(12c) $${\tilde{x}}_{t}={\tilde{U}}^{⊺}\;{\hat{x}}_{t}$$

where $\tilde{A}\in R^{S\times S}$ and $\tilde{B}\in R^{S\times l}$. This allows us to form the reduced LDS as


(13a) $${\tilde{x}}_{t+1}=\tilde{A}{\tilde{x}}_{t}+\tilde{B}z_{t}$$

(13b) $${\hat{x}}_{t}=\tilde{U}{\tilde{x}}_{t}$$

Here, the initial state ${\tilde{x}}_{1}$ is derived directly from data as ${\tilde{x}}_{1}={\tilde{U}}^{⊺}x_{1}$.

### 2.4 Parametric dynamic mode decomposition with control

For the remainder of the manuscript, we will use an index-based notation. Using the data as an example, $x_{t,i,d}\in R^{M\times 1}$ represent the column vector of the metabolite postprandial response at the *t*:th time point, *i*:th individual, and *d*:th diet. We use the colon notation (:) to indicate that a range of time points are selected resulting in the matrix $x_{1:T,i,d}\in R^{M\times T}$ and the ∗ to indicate the concatenation of responses of *D* diets for the *i*:th individual as $X_{i,*}\in R^{T\times \left(M\cdot D\right)}$. Finally, we use $X^{\left(\mathrm{tot}\right)}\in {\mathbb{R}}^{M\times \left(T\cdot D\cdot I\right)}$ to denote all data in the dataset concatenated as $X^{\left(\mathrm{tot}\right)}=\left[X_{i=1,*},X_{i=2,*}\ldots X_{i=17,*}\right]$. Parametric DMD with control (pDMDc) can make use of higher order tensor data, such as cross-over interventions, where 3-way data ($R^{I\times M\times T}$) are collected for multiple interventions into 4-way data ($R^{D\times I\times M\times T}$) (24). Thus, pDMDc has the potential to incorporate several provocations of the metabolic system into the same dynamic model, predict metabolite response to a new diet, and also identify groups of differential responders by clustering the individual latent state trajectories. Using pDMDc on the tensor data as described in Section 2.1, we can obtain system distinct matrices ${\tilde{A}}_{i}$ and input matrices ${\tilde{B}}_{i}$ per individual $i=\left\{1,2,\ldots ,17\right\}$ based on data from all diets in the dataset.

We do this by concatenating data from all diets, forming the new snapshot matrices $X_{i,*}$, $X_{i,*}^{\prime }$ and $\Omega_{i,*}$ per individual (analogous to Equations 3, 4, 8) according to Equations 14, 15, and Equation 16


(14) $$X_{i,*}=\;\left[\underset{\begin{array}{c} \mathrm{diet}\;1 \end{array}}{\underset{︸}{\begin{array}{ccccc} |\; & \;|\; & \;|\; & & \;|\;\\ x_{1,i,d=1} & x_{2,i,d=1} & x_{3,i,d=1} & \ldots & x_{T-1,i,d=1}\\ \;|\; & \;|\; & \;|\; & & |\; \end{array}}}\;\ldots \;\underset{\begin{array}{c} \mathrm{diet}\;D \end{array}}{\underset{︸}{\begin{array}{ccccc} |\; & \;|\; & \;|\; & & \;|\\ x_{1,i,d=D} & x_{2,i,d=D} & x_{3,i,d=D} & \ldots & x_{T-1,i,d=D}\\ |\; & \;|\; & \;|\; & & \;| \end{array}}}\right]$$

(15) $$X_{i,*}^{\prime }=\;\left[\underset{\begin{array}{c} \underset{\cdot }{\;}\\ \mathrm{diet}\;1 \end{array}}{\underset{︸}{\begin{array}{ccccc} |\; & \;|\; & |\; & & |\\ x_{2,i,d=1} & x_{3,i,d=1} & x_{4,i,d=1} & \ldots & x_{T,i,d=1}\\ \;|\; & |\; & |\; & & \;|\; \end{array}}}\;\ldots \;\underset{\begin{array}{c} \mathrm{diet}\;D \end{array}}{\underset{︸}{\begin{array}{ccccc} |\; & \;| & \;|\; & & \;|\\ x_{2,i,d=D} & x_{3,i,d=D} & x_{4,i,d=D} & \ldots & x_{T,i,d=D}\\ \;| & \;| & \;|\; & & \;| \end{array}}}\right]$$

(16) $$\Omega_{i,*}=\left[\underset{\begin{array}{c} \mathrm{diet}\;1 \end{array}}{\underset{︸}{\begin{array}{ccccc} | & | & | & & |\\ x_{1,i,d=1} & x_{2,i,d=1} & x_{3,i,d=1} & \ldots & x_{T-1,i,d=1}\\ | & | & | & & |\\ z_{1,d=1} & 0 & 0 & \ldots & 0 \end{array}\;}}\ldots \;\underset{\begin{array}{c} \mathrm{diet}\;D \end{array}}{\underset{︸}{\begin{array}{ccccc} | & | & | & & |\\ x_{1,i,d=D} & x_{2,i,d=D} & x_{3,i,d=D} & \ldots & x_{T-1,i,d=D}\\ | & | & | & & |\\ z_{1,d=D} & 0 & 0 & \ldots & 0 \end{array}}}\right]$$

Subsequently, we proceed with DMDc, completely analogous to Equations 9–12a, 12b, 12c but substituting $X^{\prime }$ (Equation 4) with $X_{i,*}^{\prime }$ (Equation 15) and Ω (Equation 8) for $\Omega_{i,*}$ (Equation 16), to obtain the matrices ${\tilde{A}}_{i}$, ${\tilde{B}}_{i}$ and ${\tilde{U}}_{i,d}$. For a detailed derivation, see the Supplementary material, Section 1. This gives the following system, analogous to Equations 13a, 13b:


(17a) $${\tilde{x}}_{t+1,i,d}={\tilde{A}}_{i}\;{\tilde{x}}_{t,i,d}+{\tilde{B}}_{i}\;z_{t,d}$$

(17b) $${\hat{x}}_{t,i,d}={\tilde{U}}_{i,d}{\tilde{x}}_{t,i,d}\text{.}$$

Here the dimensions of the system are the same as for Equations 13a, 13b and the indices *t*, i, and d represent time, individual, and diet, respectively. The initial state of each individual and diet ${\tilde{x}}_{1,i,d}$ is derived as ${\tilde{x}}_{1,i,d}={\tilde{U}}_{i,d}^{⊺}x_{1,i,d}$.

When using Equations 17a, 17b to predict the response to a new diet, we avoid projecting the states onto a lower-dimensional subspace (corresponding to setting *S* = *M* during the SVD of the snapshot matrix $X_{i,*}^{\prime }$ and thus effectively omitting ${\tilde{U}}_{i,d}$ from Equations 17a, 17b) but still rank-reducing the system (see Supplementary material Equation S8). This is done to reduce prediction errors since the interpretability of the model is not the focus.

To identify metabotypes, we take an alternative approach to deriving the LDS, using a mapping between the full and latent system that is shared by all individuals, thereby making state trajectories comparable between individuals. We start by forming the matrix $X^{\left(\mathrm{tot}\right)}\in R^{M\times \left(T\cdot D\cdot I\right)}$ by column-wise concatenation of data for all individuals, time points, and diets. We then perform an SVD of $X^{\left(\mathrm{tot}\right)}$= $U^{\left(\mathrm{tot}\right)}\Sigma^{\left(\mathrm{tot}\right)}W^{\left(\mathrm{tot}\right)⊺}$ and truncate the resulting matrices using $S\leq \mathrm{rank}\left(X^{\left(\mathrm{tot}\right)}\right)$ components to obtain the matrices ${\tilde{U}}^{\left(\mathrm{tot}\right)}\in {\mathbb{R}}^{M\times S}$, ${\tilde{\Sigma }}^{\left(\mathrm{tot}\right)}\in {\mathbb{R}}^{S\times S}$ and ${\tilde{W}}^{\left(\mathrm{tot}\right)}\in R^{\left(T\cdot D\cdot I\right)\times S}$. Now ${\tilde{U}}^{\left(\mathrm{tot}\right)}$ defines the mapping between the full system and a latent, reduced system, shared by all individuals. Next, we use ${\tilde{U}}^{\left(\mathrm{tot}\right)}$ to project the snapshot matrices $X_{i,*}$ and $X_{i,*}^{\prime }$ onto this latent space, forming two new matrices according to


(18a) $${\tilde{X}}_{i,*}={\tilde{U}}^{\left(\mathrm{tot}\right)⊺}X_{i,*}$$

(18b) $${\tilde{X}}_{i,*}^{\prime }={\tilde{U}}^{\left(\mathrm{tot}\right)⊺}X_{i,*}^{\prime }$$

Similar to Equation 16, we then form a matrix ${\tilde{\Omega }}_{i,*}$according to


(19) $${\tilde{\Omega }}_{i,*}=\left[\underset{\begin{array}{c} \mathrm{diet}\;1 \end{array}}{\underset{︸}{\begin{array}{ccccc} | & | & | & & |\\ {\tilde{X}}_{1,i,d=1} & {\tilde{X}}_{2,i,d=1} & {\tilde{X}}_{3,i,d=1} & \ldots & {\tilde{X}}_{T-1,i,d=1}\\ | & | & | & & |\\ z_{1,d=1} & 0 & 0 & \ldots & 0 \end{array}\;}}\ldots \;\underset{\begin{array}{c} \mathrm{diet}\;D \end{array}}{\underset{︸}{\begin{array}{ccccc} | & | & | & & |\\ {\tilde{X}}_{1,i,d=D} & {\tilde{X}}_{2,i,d=D} & {\tilde{X}}_{3,i,d=D} & \ldots & {\tilde{X}}_{T-1,i,d=D}\\ | & | & | & & |\\ z_{1,d=D} & 0 & 0 & \ldots & 0 \end{array}}}\right]$$

Finally, using ${\tilde{X}}_{i,*}^{\prime }$ and${\tilde{\Omega }}_{i,*}$ from Equations 18a, 18b, 19, we again proceed with DMDc according to Equations 9–12a, 12b, 12c, but this time substituting the snapshot matrix $X^{\prime }$ (Equation 4) with ${\tilde{X}}_{i,*}^{\prime }$ (Equations 18a, 18b) and Ω (Equation 8) with ${\tilde{\Omega }}_{i,*}$ (Equation 19), to obtain the matrices ${\tilde{A}}_{i}$ and ${\tilde{B}}_{i}$. For a detailed derivation, see the Supplementary Material, Section 2. Together with the matrix ${\tilde{U}}^{\left(\mathrm{tot}\right)}$, this allows us to write the system as, in Equations 20a and 20b


(20a) $${\tilde{x}}_{t+1,i,d}={\tilde{A}}_{i}\;{\tilde{x}}_{t,i,d}+{\tilde{B}}_{i}\;z_{t,d}$$

(20b) $${\hat{x}}_{t,i,d}={\tilde{U}}^{\left(\mathrm{tot}\right)}\;{\tilde{x}}_{t,i,d}$$

Here, the three diets are given distinct initial states ${\tilde{x}}_{1,i,d}={\tilde{U}}^{\left(\mathrm{tot}\right)⊺}x_{1,i,d}$ and initial inputs $z_{1,d}$ to the same individual LDS given by the system matrix ${\tilde{A}}_{i}$ and input matrix ${\tilde{B}}_{i}$.

### 2.5 Predicting postprandial metabolite response to unseen diets

Prediction of response to unseen diets with pDMDc was done using measured data (Section 2.1.1) and simulated data (Section 2.1.2), where evaluation of performance was measured using *R*^2^ on test sets.

Pooling of the measured data was done using the responses to the three dietary exposures for all individuals (*n* = 17), thus each response per individual is considered one “observation.” Using the measured data, a training, validation, and test split of 60, 20, and 20% of the observation were used, respectively. To estimate the *R*^2^ on the entire dataset, the order of observations in the entire dataset was randomly permuted prior to splitting, training, and evaluation. This resampling without replacement was performed 100 times and the average *R*^2^ from all iterations was calculated as the final one.

To investigate if the inclusion of further dietary exposures hold the potential to improve the predictions, initially the same pooling of the data (Section 2.1.2) from 17 simulated individuals was performed. However, instead of using the same split as for the measured data we assigned a large test set of the response to 50 diets on 17 individuals and gradually increased the number of responses to diets in the training and validation set from 3 up to 40 diets.

Model complexity was chosen using the residuals of the training and validation data. When the root mean squared error (RMSE) of the validation data deviated more than 30% from the RMSE on the training data, the training stopped and the model that achieved the lowest RMSE was chosen. Model performance evaluation was measured using *R*^2^-values computed according to Equation 21.


(21) $$R^{2}=1-\frac{\sum_{i}^{N}{\left(vec{\left(\hat{X}\right)}_{i}-vec{\left(X\right)}_{i}\right)}^{2}}{\sum_{i}^{N}{\left(\bar{X}-vec{\left(X\right)}_{i}\right)}^{2}}$$

where $vec\left(X\right)$ represents the vectorized tensor X (entire test set) and $\hat{X}$ (entire predicted test set) denotes the predicted tensor while $\bar{X}$ denotes the average of all the values N in the tensor.

### 2.6 Metabotyping

Metabotyping was performed using both pDMDc and CP (representing a state-of-the-art decomposition method) on the measured data from the intervention study described in Section 2.1.1 and on the simulated data described in Section 2.1.2. To validate the use of pDMDc for metabotyping, we aimed to identify two previously published metabotypes (15) in the measured data. Using simulated data, metabotyping was performed to identify ground truth clusters of diabetic and healthy individuals as described in Section 2.1.2.

To identify metabotypes using pDMDc, we compared the resulting latent state trajectories from pDMDc (Section 2.4) between individuals by taking the pairwise cosine similarities $c_{s,i,j,d}$ and arranging them in a square matrix with I=17 rows and columns as in Equation 22:


(22) $$c_{s,i,j,d}=\frac{{\tilde{x}}_{s,1:T,i,d}{\tilde{x}}_{s,1:T,j\neq i,d}^{⊺}}{\;||{\tilde{x}}_{s,1:T,i,d}||\;||\;{\tilde{x}}_{s,1:T,j\neq i,d}||}$$

Here, ${\tilde{x}}_{s,1:T,i,d}\in R^{1\times T}$ denotes the latent state trajectories, the indices i and j represent distinct individuals producing a square similarity matrix $c_{s,1:I,1:I,d}\in R^{I\times I}$ per state and diet. We proceeded by clustering the matrix using agglomerative clustering to identify groups of individuals with similar metabolite dynamics per state. In the case of measured and simulated data, the number of clusters to search for was known.

Model complexity in pDMDc for metabotyping, i.e., the choice of S and V (for simplicity we choose S=V), was assessed by identifying the inflection point in the scree plot of the singular values derived from the SVD $X^{\left(\mathrm{tot}\right)}$= $U^{\left(\mathrm{tot}\right)}\Sigma^{\left(\mathrm{tot}\right)}W^{\left(\mathrm{tot}\right)⊺}$ (28, 29). Further, we classified the Smost dominant dynamical profile by clustering the covariance matrix $C\in R^{M\times M}$ of the measured data for each metabolite averaged over all individuals and diets $\bar{\bar{X}}\in {\mathbb{R}}^{M\times T}$ (Equations 23, 24). The dominant dynamical metabolite profiles were compared with latent state trajectories ${\tilde{x}}_{s,1:T,i,d}$ using correlation.


(23) $$\bar{\bar{X}}=\frac{1}{I\;D}\overset{I}{\underset{i=1}{\sum }}\;\overset{D}{\underset{d=1}{\sum }}\;x_{1:M,1:T,i,d}$$

(24) $$C=cov\left(\bar{\bar{X}}\right)$$

Clusters of metabolites in the covariance matrix were identified using agglomerative clustering with “Farthest distance” as method and one minus the sample correlation between points as distance metric in MATLAB (Statistics and Machine Learning Toolbox, version 7.10.0, R2022a, The MathWorks, Inc., Natick, Massachusetts, US).

The potential metabotype clusters identified using pDMDc were compared to clusters derived from the scores of the unconstrained CP (30), representing a state-of-the-art tensor decomposition method (Equation 25).


(25) $$X=\overset{F}{\underset{f=1}{\sum }}r_{f}⊗q_{f}⊗p_{f}⊗h_{f}$$

Here $r\in R^{M}$, $q\in R^{T}$, $p\in R^{I}$, and $h\in R^{D}$ denote factors representing the modes (metabolites, time points, individuals, and diets respectively) of the tensor $X\in R^{M\times T\times I\times D}$. The factors are multiplied using the outer product ⊗ and form a tensor with four axes/modes and the *F* components are summed to reconstruct the tensor X. This is similar to PCA where the factors of different components are constrained to be orthogonal to each other and the tensor is constrained to only two axes, i.e., a matrix. We refer to p as individual scores, and the other factors as metabolite, time, and diet loadings (r, q, and h respectively). The data for each metabolite was scaled to unit standard deviation (31). The CP models were derived using the MATLAB N-way toolbox (32) and model complexity was chosen by selecting the model that explained the most variance without exhibiting two-factor degeneracies.

Using CP, clusters were identified using k-means on the individual scores $p_{f}$ and compared to clusters identified using pDMDc. Agglomerative clustering was performed on the similarity matrix derived using pDMDc since this clustering performs well on matrices similar to covariance matrices while the k-means was chosen when clustering CP scores, as it is a common method for clustering matrices with no particular structure (27). To assess the biological relevance of the clusters, we used ANOVA to find associations between clusters and clinical baseline and anthropometric measures. For strong associations (*p* < 0.05) with a plausible biological relationship between metabolites, clusters, and baseline measures, we infer that the clusters may correspond to potential metabotypes.

To compare variance captured using pDMDc and CP we calculated the correlation between orthonormal basis vectors ${\tilde{U}}_{1:M,s}^{\left(\mathrm{tot}\right)}$ and the metabolite loadings $r_{f}$. Furthermore, we calculated correlation between the latent state trajectories ${\tilde{x}}_{1:T,i,d}$ and the time loadings $q_{f}$ to assess the dynamic behavior captured in each component and state. The trajectories and time loadings were compared to mean metabolite trajectories of grouped metabolites from clustering the data covariance matrix using correlation (Equation 24).

## 3 Results

### 3.1 Predicting postprandial metabolite response to unseen diets

**Figure 3:**
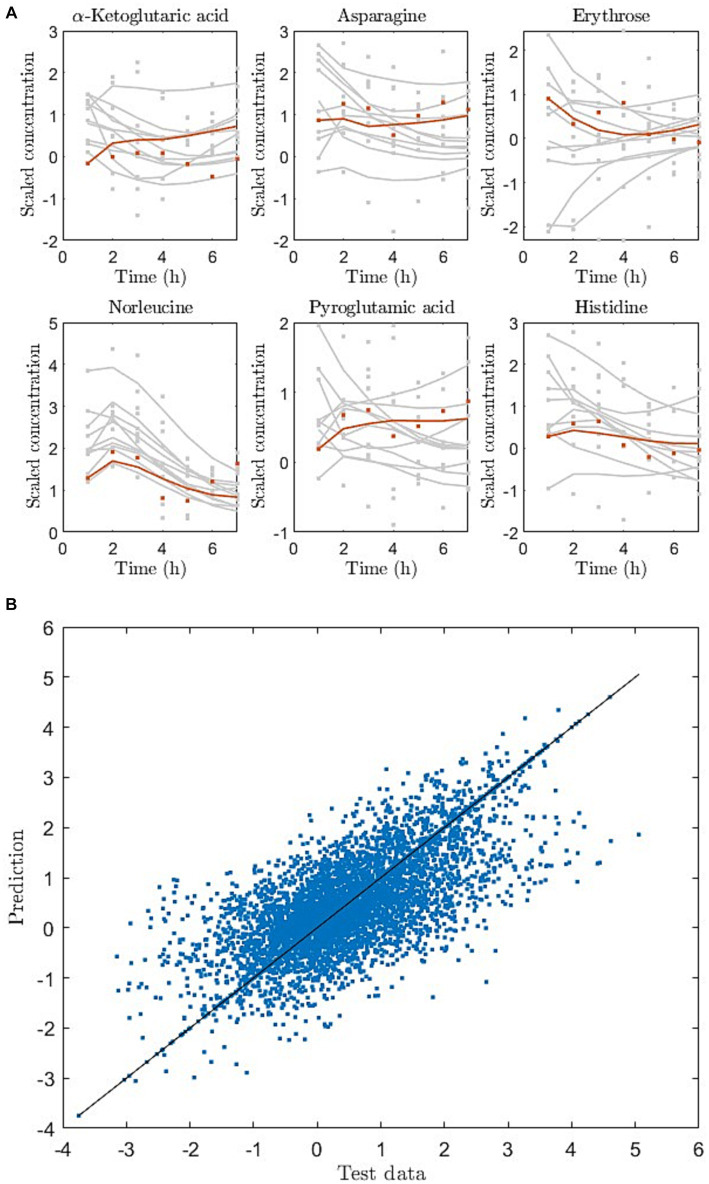
**(A)** Dynamic metabolite trajectories for training (gray) and holdout test (red) observations, exemplified for 6 out of 79 metabolites. Here dots are data and lines are model prediction (red) or reconstruction (grey). **(B)** Entire test data and prediction of test data as scatter plot for one of the cross-validation iterations. The line represents the perfect match between data and predictions.

Using the baseline metabolite measurement and the dietary information (macronutrient amounts) we predicted the response of unseen diets in the measured data. Since the participants (*n* = 17) were only exposed to three meal challenges in the study, we pooled data from all individuals and considered each individual with their metabolite response to the three challenges as three “observations.” Using the pooled data we were able to predict the test sets (10 observations) with 40% explained variance (*R*^2^ = 0.4) (averaged over all resampling iterations). Figure 3A shows a predicted response to a unseen dietary exposure in six of the 79 metabolites, whereas Figure 3B shows the prediction and data of the entire test set as a scatter plot.

**Figure 4:**
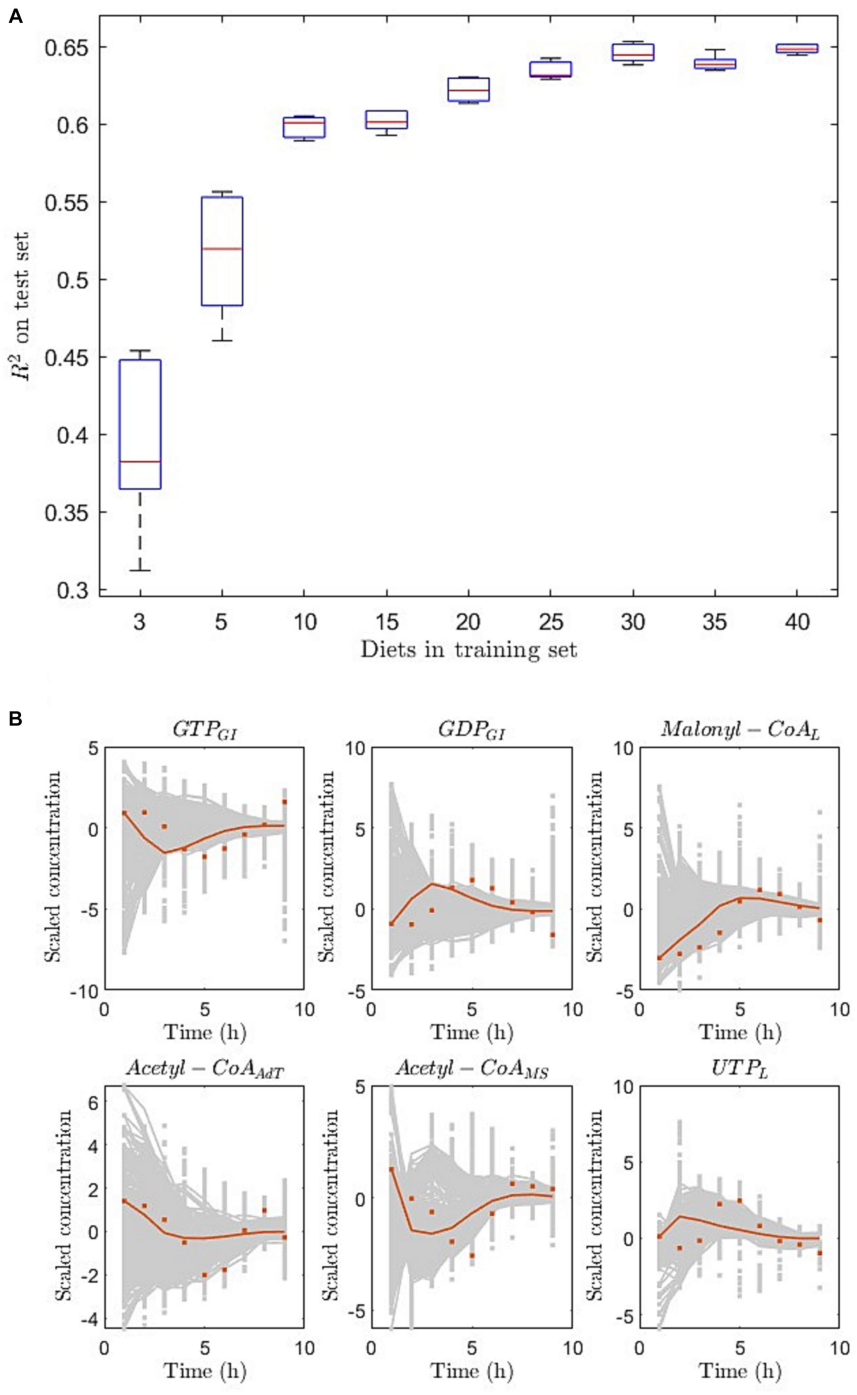
Prediction of responses to simulated diets using pooled individuals as in Figure 3. **(A)** Prediction metric R2 of a large test set with increasing number of diets in the training set (5 iterations of scrambling the examples prior to splitting training and validation). **(B)** Prediction of test example (red) and training examples (grey) shown in six metabolite (out of 130 in total) responses. Here dots are data and lines are model prediction (red) or reconstruction (grey). The predictions are made using 40 diets in the training set. The subscripts GI,L, AdT, and MS stand for metabolites modelled in gastrointestinal tract, liver, adipose tissue, and skeletal muscle, respectively.

Using simulated data from 17 individuals (pooled data as in the measured data), we observed that increasing the number of exposures to diets in the training set increased *R*^2^ on the large test set (170 examples and 50 diets) shown in Figure 4. When increasing the number of diets successively from 3 to 40, *R*^2^-values displayed a corresponding increase from around 0.38 to 0.65 (Figure 4). As input to the pDMDc model, we used the simulated metabolite baseline and the dietary information (glucose and triglyceride content and their delay coefficients explained in Section 2.1.2).

### 3.2 Metabotyping

Metabotyping was performed in measured data (Section2.1.1) to identify already published metabotypes and on simulated data (Section 2.1.2) to identify ground truth clusters of healthy and diabetic individuals.

**Figure 5:**
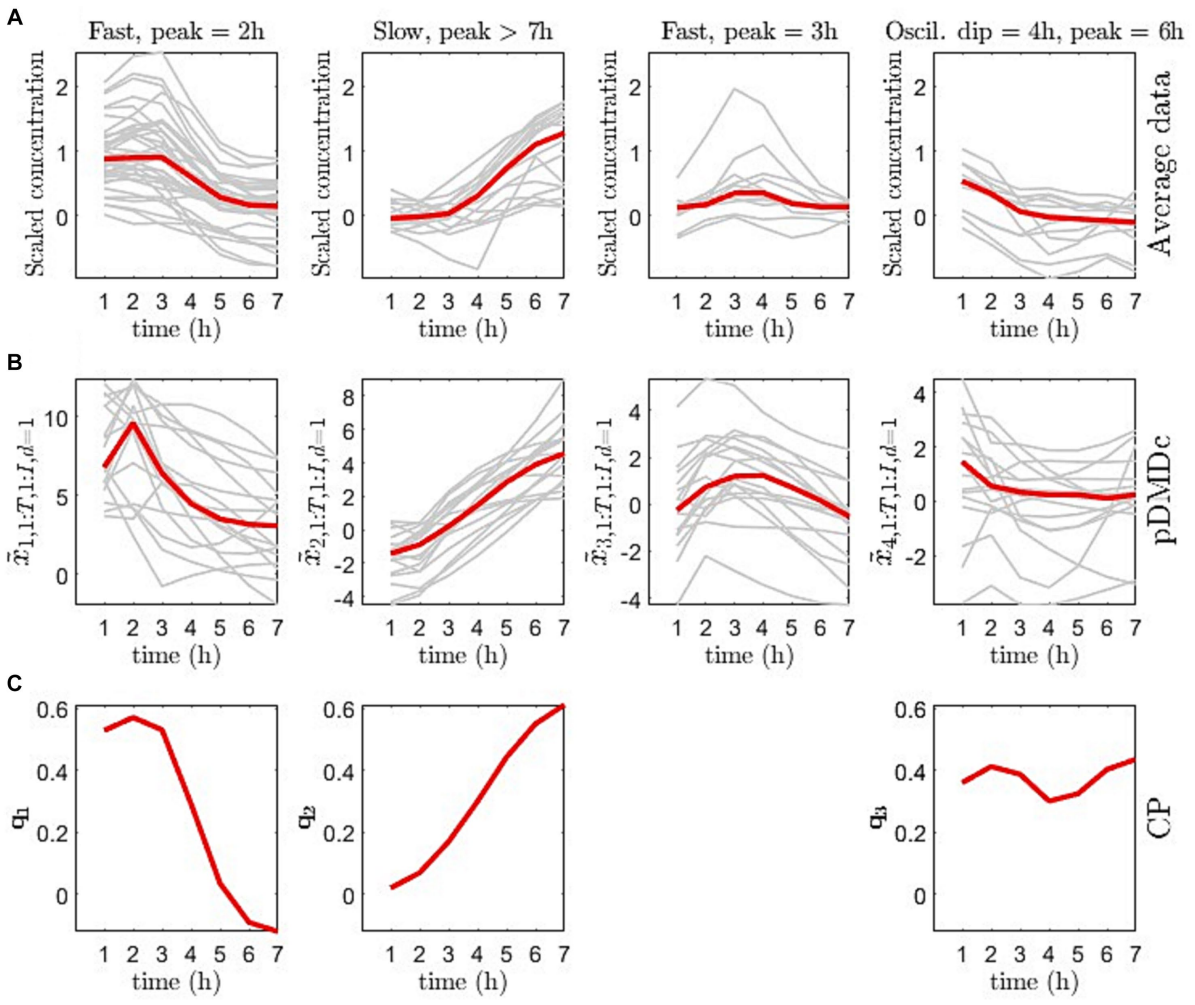
**(A)** Average measurement trajectory per metabolite (grey lines) and mean measurement trajectory per dynamical class (red line) obtained from clustering of the covariance matrix of the averaged data per metabolite. **(B)** Individual state trajectories for the pickled herring diet (grey lines) and median of individual trajectories per state (red line). **(C)** CP time loadings (q from Equation 23) for the 3-component CP model.

The scree plot of the singular values (used to choose model complexity) resulted in an inflection point at four SVD components (data not shown), i.e., four latent pDMDc states. The correlation between averaged metabolite clusters and latent states was calculated to identify what metabolite category the states represented. The first state trajectories captured metabolite trajectories peaking at 2 h post ingestion (ρ = 0.88), the second captured slower dynamics, peaking at >7 h (ρ = 0.97), the third state captured oscillatory dynamics (ρ = 0.85), and the fourth state captured intermediate dynamics (ρ = 0.93), peaking at 3 h (Figures 5A,B). Using CP, only three components could be extracted that showed both interpretable dynamics and biologically relevant clusters in scores (Figure 5C). CP models using more than 3 components had degenerate solutions in accordance with previous findings from this data set (15). The CP time loadings reflected pDMDc states one (ρ = 0.90) and two (ρ = 0.99), but did not capture dynamics of state three (ρ = −0.06) and four (ρ = −0.54). Regularization of the metabolite mode was attempted, but clustering in scores revealed no relevant biological connection to baseline clinical parameters (data not shown).

**Figure 6:**
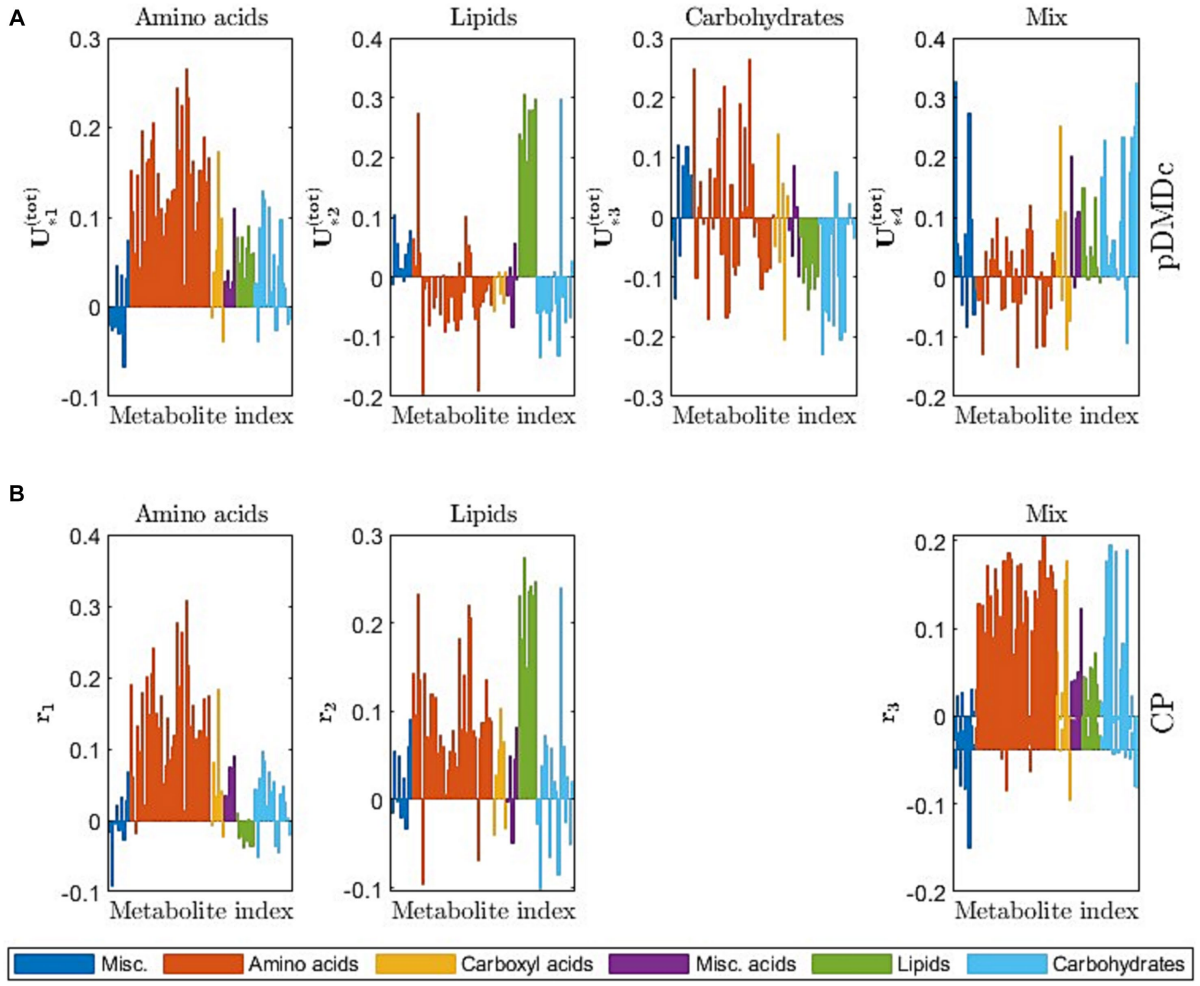
**(A)** Column vectors of ${\tilde{U}}^{\left(\mathrm{tot}\right)}$color-coded by metabolite category, describing the contribution of metabolites (similar to PCA loadings) to the observed states. **(B)** CP metabolite loadings (r from Equation 25) color-coded by metabolite category, describing the metabolite contribution to each CP component.

Figure 6 gives a visual overview of the metabolite contribution to each state in pDMDc and components in CP (Figure 5), via column vectors of the shared output matrix ${\tilde{U}}^{\left(\mathrm{tot}\right)}$ (Section 2.4) and metabolite loading vectors (Section 2.5). The figure shows that large groups of metabolites (Figure 6) coincide with dynamic profiles (Figure 5A) in each given latent state and component of the models. The CP metabolite loadings (Figure 6C) coincide well in the first (ρ = 0.89) and second (ρ = 0.78) component with the first and second pDMDc state but states three and four correlated more poorly in the third component (ρ = −0.41 and ρ = −0.44, respectively). For the metabolite data used in this study, the first state was represented predominantly by amino acids, which had an early absorption peak at around 2 h (Figure 6, first column). The second state was represented predominantly by lipids (Figure 6, second column), with a late peak after 7 h (Figure 5, second column). The third state was represented predominantly by carbohydrates (Figure 6A, third column), with an oscillatory dynamic behavior. The fourth state was represented by a mix of carbohydrates, carboxyl acids, and amino acids among others (Figure 6A, fourth column), with a fast dynamic behavior (peak around 3 h).

**Figure 7:**
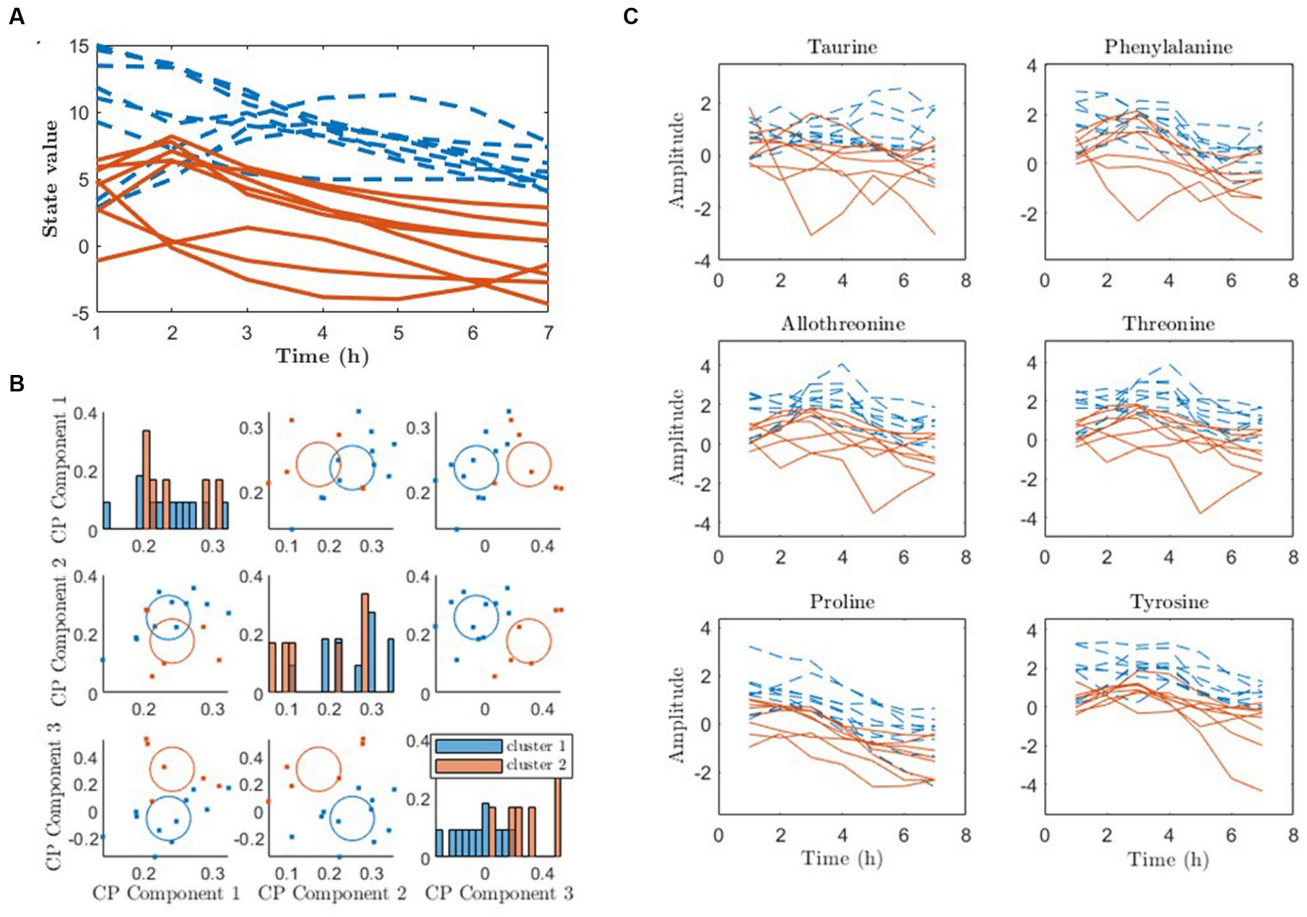
Clustering of metabolic response to diet using pDMDc and CP to infer metabotypes (red and blue lines and dots). **(A)** The individual state trajectories of the first state in response to the meat diet, using four latent states. **(B)** K-means clustering of CP scores. **(C)** Raw data of amino acids contributing most strongly to the first column vector of ${\tilde{U}}^{\left(\mathrm{tot}\right)}$, color-coded according to the clustering.

The state trajectories ${\tilde{x}}_{i,d}$ obtained from the 4-state pDMDc model were clustered state by state using agglomerative clustering on the cosine similarities between individual trajectories as described in Section 2.5. As shown in Figure 7A, clustering in the first state trajectories in the meat diet produced two response patterns, one with mostly positive trajectories (blue) and mostly negative trajectories (red). Using the 3-component CP model similar clusters were found with 94% overlap to the ones found with pDMDc (Figure 7B). However, a 100% match could be found using two-components but explaining less variance of the data (15). ANOVA analysis revealed that the clustering was significantly associated with baseline creatinine levels (*p* = 0.007). Finally, the amino acids that contributed the most to the first state showed differences in response between clusters (Figure 7C).

**Figure 8:**
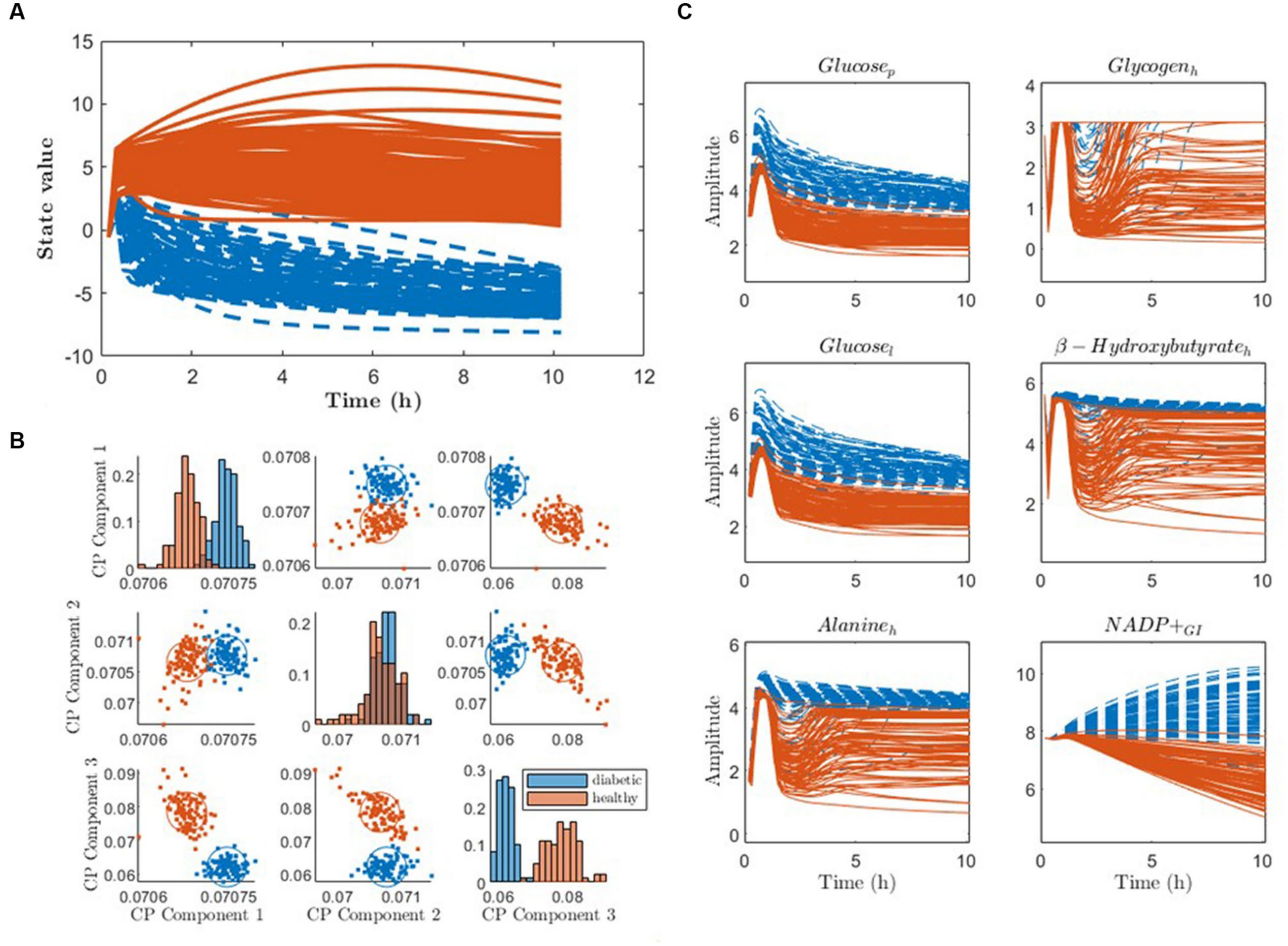
Clustering of state trajectories and CP scores to identify the ground truth simulated diabetic (blue) and healthy (red) patients. **(A)** Individual DMD state trajectories of the fourth state using four latent states. **(B)** CP scores clustered using the three-component model. **(C)** Raw simulated data of metabolites with the strongest contributors to the fourth column vector of ${\tilde{U}}^{\left(\mathrm{tot}\right)}$, color-coded according to the clustering. The subscripts p, h, l, and GI stand for metabolites modelled in plasma, heart, liver, and gastrointestinal tract, respectively.

When applying pDMDc and CP for metabotyping on simulated data we were able to identify simulated diabetic and healthy individuals (Figure 8). Using pDMDc, the scree plot showed inflection point at four states (data not shown) and diabetic clusters were identified in states two and three but were separated most clearly in state four (Figure 8A). Although ground truth clusters could also be found using CP (Figure 8B), more than three components could not be modelled without factor degeneracy even though clustering of the covariance matrix revealed dynamics which pDMDc could model but not CP (data not shown). The most dominating metabolites in the fourth state displayed clear cluster separation (Figure 8C).

## 4 Discussion

In this work, we explored combining two variants of DMD (DMD with control and parametric DMD) for the purpose of prediction and classification of metabolomic data from multiple dietary challenges. We demonstrated the utility of the method in two use cases: (i) predicting measured and simulated and postprandial response to new diets based on data from prior dietary interventions (ii) identifying metabotypes in metabolomic data from a dietary intervention crossover study and simulated data. We show that it is possible to predict responses to new dietary exposures using pooled measured and simulated data. Additionally, we showed that prediction performance is increased when adding more diets to the data available for training the DMD model. Furthermore, we showed that metabotyping can be performed using the same pDMDc model, and that the clustering results are comparable to those produced by the state-of-the-art method CP. To our knowledge, this study represents the first application of DMD to metabolite data.

Using pDMDc to predict the simulated data set from the virtual human metabolic model, we showed that the predicted variance could be improved significantly by adding more diets (Figure 4). This was done since no more than three dietary exposures were available in the measured data. The result indicates that using more dietary exposures from dietary intervention studies could improve predictive performance, and that the pDMDc algorithm could be useful for predicting large parts of the measurable metabolism with high precision. However, the efficacy needs to be confirmed in large-scale multi-diet intervention studies.

We showed that pDMDc is interpretable in terms of what metabolite dynamics each state represents (Figures 5, 6), in contrast to deep learning methods that often lack an explicit representation of metabolite dynamics, often making interpretation difficult (33). Furthermore, pDMDc is linear and built on well-understood mathematics used in many fields of physics and mathematics, including linear control theory. Thus, pDMDc provides the potential to approach PN by estimating the optimal dietary input given a target metabolite trajectory (18). This could potentially help find the best personalized diet in a population with differential responders. To do this, a multi-diet intervention would be needed for the model to learn the individual responses in a large set of metabolites to varied diets. Thereafter, *a priori* known target levels of important biomarkers would be set and used together with the trained model to calculate what dietary input is required to meet those levels. This is analogous to existing methodology in automatic control, which is well understood and widely applied to LDSs. While this is a relatively easy problem in cases where we have perfect knowledge of the system under study, it should be noted that pDMDc only provides an approximation to the metabolic system based on a limited number of postprandial responses. It is therefore very important that the dietary challenges are as varied as possible, to capture as much of the systems capabilities as possible. Other machine learning methods, like random forest and network models, are not as well studied in the context of automatic control, and LDSs are preferred when applicable (18, 34). Additionally, the proposed pDMDc methodology uses differences in model states (roughly corresponding to groups of metabolites) to identify metabotypes. This means that it naturally highlights metabolites that could potentially be used as biomarkers for metabotyping, as well as giving suitable time points for measuring these metabolites after a dietary provocation.

Metabotypes were successfully identified with pDMDc in measured and simulated data by clustering latent state trajectories derived using a shared output map ${\tilde{U}}^{\left(\mathrm{tot}\right)}$ (Equations 18a, 18b). Imposing the shared output map makes the individual state trajectories comparable, enabling clustering of individuals which to our knowledge has not been done before using this methodology. The high correlations (Section 3.2) between trajectories in data (Figure 5A), latent states (Figure 5B), and CP time loadings (Figure 5C) show that pDMDc can be an interpretable model in terms of visually inspecting what each latent state represent. This is further supported in that metabolite contribution to each latent state can be identified in the output map ${\tilde{U}}^{\left(\mathrm{tot}\right)}$.

Metabotype clusters of the cosine similarity scores overlapped well with clusters derived from CP in already published metabotypes (15). However, using simulated data ground truth clusters (healthy and diabetics) were identified with highest classification accuracy in the fourth pDMDc state, while they were identified in all three CP components. This indicates that CP may be superior to pDMDc when it comes to clustering. Furthermore, CP has the advantage of enabling an overview of the data in one plot (subplots of factors per component (35)). In contrast, latent pDMDc states must be clustered per state and per dietary exposure and a full overview of the compressed data using the model is not trivial to produce. However, unconstrained CP can degenerate in spite of not all dynamics from the data being accounted for in the CP model. This problem is not present using pDMDc since model complexity depends on the SVD which does not degenerate due to orthogonal components. Although divergent predictions can occur in DMD model due to instability of the LDS, this can be mended by scaling eigenvalues (20). To avoid degeneracy problems, constraints on the CP model can be imposed, such as orthogonality constraints on the metabolite mode making it more similar to the orthonormal basis ${\tilde{U}}^{\left(\mathrm{tot}\right)}$ in pDMDc. However, in the present study we focused on comparing our method to unconstrained CP, as it is unique up to permutation and scaling ambiguities, which is not the case when imposing constraints (31, 35).

When metabotyping performed on static markers and related to disease outcomes, which is often the case (11), it is far from certain that individuals within the same metabotype will respond similarly to targeted food. Conversely, identifying differential responders to meals prior to relating them to outcomes, as is done using pDMDc, may increase the likelihood that the individuals within each identified group respond similarly to a tailored diet. Indeed, the more diets the individuals are exposed to, the more likely it is that the groups of differential responders represent true metabotypes, since more information of their metabolic system is uncovered. An added advantage of pDMDc is that it allows clustering of differential responders and prediction of response to new diets based on the same model, contrary to purely predictive or descriptive models like random forests and CP.

Limitations include that to our knowledge there is currently no well-established method for forecasting multivariate metabolite time series using baseline values and dietary information. Consequently, no comparison to the state-of-the-art was possible in the prediction case. Additionally, the dietary inputs were macronutrients, representing a highly simplified encoding of the nutritional content of the meals. More informative encodings, e.g., included food items and type of food preparation, might improve prediction and clustering results. Furthermore, the measured dataset only included 17 individuals each with 3 dietary challenges, hampering generalization of results from this part of the study. Finally, no ground truth metabotype clusters were available using the measured data, yielding a proxy validation by identifying previously published clusters.

## 5 Conclusion

We have developed a method for analysis of time-resolved metabolomics data, based on a combination of parametric DMD and DMD with control for analyzing time-resolved omics tensor data. To the best of our knowledge, this is the first use of DMD as a tool for clustering and prediction in metabolomics. The method was applied to measured and simulated data to predict metabolite responses of unseen dietary exposures using the metabolite baseline and the dietary information. Using simulated data, we showed that metabolite responses to dietary provocation could be predicted with high accuracy. Additionally, the pDMDc model was used to infer metabotypes from experimental and simulated data by clustering LDS state trajectories, resulting in agreement with previously published outcomes, although CP was shown to be a stronger metabotyping tool. The method thus has strong potential for PN applications as it learns the relationship between dynamic postprandial response and dietary exposures. Furthermore, it has the potential to provide optimal tailored diets, given target levels in biomarkers of interest. However, this potential still must be demonstrated using experimental data from large scale studies using diverse intervention diets. Even though the pDMDc methodology presented in this article was developed and applied in the context of personalized nutrition and multiple dietary exposures, it should be noted that prediction and identification of differential responders in other time-resolved omics or high dimensional biological data may also be suitable use cases. Future directions of development may include refining the estimation of the input dynamics such that it can provide a more realistic impact on the dynamics and investigating optimal dietary input for *a priori* known metabolically healthy target levels. Finally, for improved prediction performance and accuracy of pDMDc-tailored diets, a deeper investigation of which type of nutritional information that should be used as input is needed.

## Data availability statement

The data analyzed in this study is subject to the following licenses/restrictions: The data relating to this manuscript are protected under GDPR and cannot be publicly available. However, they can be provided on reasonable request. Requests to access these datasets should be directed to ann-sofie.sandberg@chalmers.se.

## Ethics statement

All study participants gave written informed consent before participating in the study. The study was approved by the regional ethical committee at the University of Uppsala and performed at Foodfiles (former KPL Good Food Practice) in accordance with the Helsinki Declaration of 1964 with later revisions. The trial was registered at clinicaltrials.gov (NCT02381613).

## Author contributions

VS: Conceptualization, Formal analysis, Investigation, Methodology, Software, Validation, Visualization, Writing – original draft, Writing – review & editing. MJ: Formal analysis, Funding acquisition, Methodology, Supervision, Writing – review & editing. CB: Conceptualization, Methodology, Supervision, Writing – review & editing. AS-S: Data curation, Resources, Writing – review & editing. RL: Funding acquisition, Supervision, Writing – review & editing. MW: Conceptualization, Investigation, Methodology, Supervision, Writing – review & editing.
