# Supplementary material for: Data-driven analysis and prediction of dynamic postprandial metabolic response to multiple dietary challenges using dynamic mode decomposition
Source: Front Nutr. 2024 Jan 12;10:1304540. doi: 10.3389/fnut.2023.1304540 (PMC10865386; doi:10.3389/fnut.2023.1304540)
Supplement: Supplementary file 1 [file Data_Sheet_1.pdf]

frontiers

# *Supplementary Material*

## S.1 Parametric Dynamic Mode Decomposition with Control for Prediction

The following section gives a detailed explanation of the derivation of pDMDc for prediction where SVD notation from Section 2.3 will be reused for reading purposes. From Section 2.3.4 we have the extended matrices which include all the diets from the dietary intervention

$$\boldsymbol{X}_{\boldsymbol{i},*} = \left[\underbrace{\begin{matrix} | & | & | & & | \\ \boldsymbol{x}_{1,i,d=1} & \boldsymbol{x}_{2,i,d=1} & \boldsymbol{x}_{3,i,d=1} & \cdots & \boldsymbol{x}_{T-1,i,d=1} \\ | & | & | & & | \end{matrix}}_{diet\ 1} \cdots \underbrace{\begin{matrix} | & | & | & & | \\ \boldsymbol{x}_{1,i,d=D} & \boldsymbol{x}_{2,i,d=D} & \boldsymbol{x}_{3,i,d=D} & \cdots & \boldsymbol{x}_{T-1,i,d=D} \\ | & | & | & & | \end{matrix}}_{diet\ D}\right] \quad (S1)$$

$$\boldsymbol{X}'_{i,*} = \left[\underbrace{\begin{matrix} | & | & | & & | \\ \boldsymbol{x}_{2,i,d=1} & \boldsymbol{x}_{3,i,d=1} & \boldsymbol{x}_{4,i,d=1} & \cdots & \boldsymbol{x}_{T,i,d=1} \\ | & | & | & & | \end{matrix}}_{diet\ 1} \cdots \underbrace{\begin{matrix} | & | & | & & | \\ \boldsymbol{x}_{2,i,d=D} & \boldsymbol{x}_{3,i,d=D} & \boldsymbol{x}_{4,i,d=D} & \cdots & \boldsymbol{x}_{T,i,d=D} \\ | & | & | & & | \end{matrix}}_{diet\ D}\right] \quad (S2)$$

$$\boldsymbol{Z}_* = \left[\underbrace{\begin{matrix} | & | & | & & | \\ \boldsymbol{z}_{1,d=1} & 0 & 0 & \cdots & 0 \\ | & | & | & & | \end{matrix}}_{diet\ 1} \cdots \underbrace{\begin{matrix} | & | & | & & | \\ \boldsymbol{z}_{1,d=D} & 0 & 0 & \cdots & 0 \\ | & | & | & & | \end{matrix}}_{diet\ D}\right] \quad (S3)$$

$$\boldsymbol{\Omega}_{i,*} = \left[\underbrace{\begin{matrix} | & | & | & & | \\ \boldsymbol{x}_{1,i,d=1} & \boldsymbol{x}_{2,i,d=1} & \boldsymbol{x}_{3,i,d=1} & \cdots & \boldsymbol{x}_{T-1,i,d=1} \\ | & | & | & & | \\ \boldsymbol{z}_{1,d=1} & \boldsymbol{0} & \boldsymbol{0} & \cdots & \boldsymbol{0} \end{matrix}}_{diet\ 1} \cdots \underbrace{\begin{matrix} | & | & | & & | \\ \boldsymbol{x}_{1,i,d=D} & \boldsymbol{x}_{2,i,d=D} & \boldsymbol{x}_{3,i,d=D} & \cdots & \boldsymbol{x}_{T-1,i,d=D} \\ | & | & | & & | \\ \boldsymbol{z}_{1,d=D} & \boldsymbol{0} & \boldsymbol{0} & \cdots & \boldsymbol{0} \end{matrix}}_{diet\ D}\right] \quad (S4)$$

.

We still have the linear relationship between the next time points $\boldsymbol{X}'_{i,*}$, the previous one $\boldsymbol{X}_{\boldsymbol{i},*}$ and dietary input $\mathbf{Z}_*$

$$\boldsymbol{X}'_{i,*} = [\boldsymbol{A}_i \quad \boldsymbol{B}_i\,] \begin{bmatrix} \boldsymbol{X}_{\boldsymbol{i},*} \\ \mathbf{Z}_* \end{bmatrix} = \boldsymbol{G}_i\, \boldsymbol{\Omega}_{i,*}. \quad (S5)$$

We now seek the best-fit solution, in the Frobenius sense, of the operator $\boldsymbol{G}$, representing a column-wise concatenation of $\boldsymbol{A}$ and $\boldsymbol{B}$. This is achieved using the truncated SVD of $\boldsymbol{\Omega}_{i,*}$ resulting in the rank-reduced matrices $\widehat{\boldsymbol{U}}_{i,*} \in \mathbb{R}^{(M+l)\times V}$, $\widehat{\boldsymbol{W}}_{i,*} \in \mathbb{R}^{(T-1)\times V}$ and $\widehat{\boldsymbol{\Sigma}}_{i,*} \in \mathbb{R}^{V\times V}$. Solving Eq. (S5) for $\boldsymbol{G}_{\boldsymbol{i}}$ using the truncated SVD yields

$$\boldsymbol{G}_i = \boldsymbol{X}'_{i,*}\, \boldsymbol{\Omega}_{i,*}{}^{\dagger} \approx \boldsymbol{X}'_{i,*} \left(\widehat{\mathbf{U}}_{i,*}\, \widehat{\boldsymbol{\Sigma}}_{i,*}\, \widehat{\boldsymbol{W}}_{i,*}{}^{\intercal}\right)^{\dagger} = \boldsymbol{X}'_{i,*}\, \widehat{\boldsymbol{W}}_{i,*}\, \widehat{\boldsymbol{\Sigma}}_{i,*}^{-1}\, \widehat{\mathbf{U}}_{i,*}^{\intercal}. \quad (S6)$$

We now retrieve the approximations $\widehat{\boldsymbol{A}}_i$ and $\widehat{\boldsymbol{B}}_i$ of the two linear operators $\boldsymbol{A}_i$ and $\boldsymbol{B}_i$ by dividing $\boldsymbol{G}_i$ into two parts:

$$\begin{aligned}[\boldsymbol{A}_i \quad \boldsymbol{B}_i] &\approx [\widehat{\boldsymbol{A}}_i \quad \widehat{\boldsymbol{B}}_i] \\ &= \left[\boldsymbol{X}'_{i,*}\, \widehat{\boldsymbol{W}}_{i,*}\, \widehat{\boldsymbol{\Sigma}}_{i,*}{}^{-1}\, \widehat{\mathbf{U}}_{i,*}{}^{(\boldsymbol{X})\intercal} \quad \boldsymbol{X}'_{i,*}\, \widehat{\boldsymbol{W}}_{i,*}\, \widehat{\boldsymbol{\Sigma}}_{i,*}{}^{-1}\, \widehat{\mathbf{U}}_{i,*}{}^{(\boldsymbol{u})\intercal}\right]\end{aligned} \quad (S7)$$

Here $\widehat{\mathbf{U}}_{i,*}{}^{(\boldsymbol{X})\intercal} \in \mathbb{R}^{M\times V}$ and $\widehat{\mathbf{U}}_{i,*}{}^{(\boldsymbol{z})\intercal} \in \mathbb{R}^{l\times V}$ represent the bases for the metabolomics data and the dietary input respectively as $\widehat{\mathbf{U}}_{i,*}^{\intercal} = \left[\widehat{\mathbf{U}}_{i,*}{}^{(\boldsymbol{X})\intercal} \quad \widehat{\mathbf{U}}_{i,*}{}^{(\boldsymbol{u})\intercal}\right]^{\intercal}$. This results in

$$\widehat{\boldsymbol{x}}_{t+1,i,d} = \widehat{\boldsymbol{A}}_i\, \widehat{\boldsymbol{x}}_{t,i,d} + \widehat{\boldsymbol{B}}_i\, \boldsymbol{z}_{t,d}. \quad (S8)$$

Projecting the system onto a smaller subspace is not done for prediction purposes, hence the system in Eq. (S8) is the final pDMDc LDS for prediction.

### S.2 Parametric Dynamic Mode Decomposition with Control for Metabotyping

To identify metabotypes we use an orthonormal basis $\widetilde{\boldsymbol{U}}^{(tot)} \in \mathbb{R}^{M\times S}$ stemming from the SVD (truncated from *M* to *S* columns) of the unfolded data tensor $\boldsymbol{X}^{(tot)} \in \mathbb{R}^{M\times(T*D*I)}$ as

$$\boldsymbol{X}^{(tot)} \approx \widetilde{\mathbf{U}}^{(tot)}\, \widetilde{\boldsymbol{\Sigma}}^{(tot)}\, \widetilde{\boldsymbol{W}}^{(tot)\intercal}. \quad (S9)$$

The output matrix $\widetilde{\boldsymbol{U}}^{(tot)}$ is shared among all individuals in the dataset such that the individual metabolic dynamics become comparable. This gives a structure similar to PCA and joint matrix factorization, where all individuals share a common loading matrix ($\widetilde{\boldsymbol{U}}^{(tot)}$) such that their score matrices ($\widetilde{\boldsymbol{x}}_{1:T,i,d}$) can be compared using, e.g., clustering. To achieve this, all the individual snapshot matrices $\boldsymbol{X}_{i,*}$ and $\boldsymbol{X}'_{i,*}$ are projected onto the same subspace as $\widetilde{\boldsymbol{X}}_{i,*} = \widetilde{\mathbf{U}}^{(tot)\intercal}\, \boldsymbol{X}_{i,*}$ and $\widetilde{\boldsymbol{X}}'_{i,*} = \widetilde{\mathbf{U}}^{(tot)\intercal}\, \boldsymbol{X}'_{i,*}$. The new $\boldsymbol{\Omega}_{i,*}$ is then formed using the projected data as:

$$\widetilde{\boldsymbol{\Omega}}_{i,*} = \left[\begin{array}{ccccccccc} | & | & | & & | & & | & | & | & & | \\ \widetilde{\boldsymbol{X}}_{1,i,d=1} & \widetilde{\boldsymbol{X}}_{2,i,d=1} & \widetilde{\boldsymbol{X}}_{3,i,d=1} & \cdots & \widetilde{\boldsymbol{X}}_{T-1,i,d=1} & & \widetilde{\boldsymbol{X}}_{1,i,d=D} & \widetilde{\boldsymbol{X}}_{2,i,d=D} & \widetilde{\boldsymbol{X}}_{3,i,d=D} & \cdots & \widetilde{\boldsymbol{X}}_{T-1,i,d=D} \\ | & | & | & & | & \cdots & | & | & | & & | \\ \underbrace{\boldsymbol{z}_{1,d=1} \quad \boldsymbol{0} \quad \boldsymbol{0} \quad \cdots \quad \boldsymbol{0}}_{diet\ 1} & & & & & & \underbrace{\boldsymbol{z}_{1,d=D} \quad \boldsymbol{0} \quad \boldsymbol{0} \quad \cdots \quad \boldsymbol{0}}_{diet\ D} \end{array}\right]. \quad (S10)$$

We then form the linear relationship between the projected data and the snapshot matrix

$$\widetilde{\boldsymbol{X}}'_{i,*} = [\breve{\boldsymbol{A}}_i \quad \breve{\boldsymbol{B}}_i] \begin{bmatrix} \widetilde{\boldsymbol{X}}_{i,*} \\ \mathbf{Z}_* \end{bmatrix} = \breve{\boldsymbol{G}}_i\, \widetilde{\boldsymbol{\Omega}}_{i,*}. \quad (S11)$$

Here $\breve{\boldsymbol{A}}_i \in \mathbb{R}^{S\times S}$ and $\breve{\boldsymbol{B}}_i \in \mathbb{R}^{S\times l}$ represent the description of the latent system. As in the previous section, the system is then solved in the least-squares sense using the Moore-Penrose inverse as we already use the projected data $\widetilde{\boldsymbol{X}}_{i,*}$ and $\widetilde{\boldsymbol{X}}'_{i,*}$

$$[\breve{\boldsymbol{A}}_i \quad \breve{\boldsymbol{B}}_i] \approx \widetilde{\boldsymbol{X}}'_{i,*}\, \widetilde{\boldsymbol{\Omega}}^{\dagger}_{i,*} = [\widetilde{\boldsymbol{A}}_i \quad \widetilde{\boldsymbol{B}}_i]. \tag{S12}$$

Here, the three diets are given distinct initial states $\widetilde{\boldsymbol{x}}_{1,i,d} = \widetilde{\boldsymbol{U}}^{(tot)\intercal}\boldsymbol{x}_{1,i,d}$ and initial inputs $\boldsymbol{u}_{1,d}$ to the same individual LDS given by the system matrix $\widetilde{\boldsymbol{A}}_i$ and input matrix $\widetilde{\boldsymbol{B}}_i$, see Eq. (S13)

$$\begin{aligned} \widetilde{\boldsymbol{x}}_{t+1,i,d} &= \widetilde{\boldsymbol{A}}_i\, \widetilde{\boldsymbol{x}}_{t,i,d} + \widetilde{\boldsymbol{B}}_i\, \boldsymbol{u}_{t,d} \\ \widehat{\boldsymbol{x}}_{t,i,d} &= \widetilde{\mathbf{U}}^{(tot)}\, \widetilde{\boldsymbol{x}}_{t,i,d}. \end{aligned} \tag{S13}$$
